# Geospatial Associations Between Tobacco Retail Outlets and Current Use of Cigarettes and e-Cigarettes among Youths in Texas

**DOI:** 10.4172/2155-6180.1000375

**Published:** 2017-10-18

**Authors:** Adriana Pérez, Lung-Chang Chien, Melissa B Harrell, Keryn E Pasch, Udoka C Obinwa, Cheryl L Perry

**Affiliations:** 1 Department of Biostatistics and Data Sciences, The University of Texas Health Science Center at Houston-UTHealth, School of Public Health, USA; 2 Department of Environmental and Occupational Health, University of Nevada, Las Vegas, School of Community Health Sciences, USA; 3 Department of Epidemiology, Human Genetics and Environmental Sciences, The University of Texas Health Science Center at Houston-UTHealth, School of Public Health, USA; 4 Department of Kinesiology and Health Education, College of Education at The University of Texas at Austin, USA; 5 Research Assistant, The University of Texas Health Science Center at Houston-UTHealth, Michael & Susan Dell Center for Healthy Living, Austin, USA; 6 Department of Health Promotion and Behavioral Sciences at UTHealth, University of Texas Health Science Center at Houston, School of Public Health, Austin Campus, USA

**Keywords:** Geospatial associations, Tobacco retail outlets, Kriging, Bayesian regression, Cigarettes, e-Cigarettes

## Abstract

**Introduction:**

To identify the geospatial association between the presence of tobacco retail outlets (TRO) around schools’ neighborhoods, and current use of cigarettes and e-cigarettes among adolescents in four counties in Texas.

**Methods:**

Students in grades 6, 8 and 10th were surveyed in their schools in 2014–2015. The schools’ addresses was geocoded to determine the presence of at least one TRO within half a mile of the school. Two outcomes were considered: past 30-day use of (a) cigarettes and (b) e-cigarettes. Bayesian structured additive regression models and Kriging methods were used to estimate the geospatial associations between the presence of TRO and use in three counties: Dallas/Tarrant, Harris, and Travis.

**Results:**

We observed a geospatial association between the presence of TRO around the schools and current use of cigarettes in the eastern area of Dallas County and in the southeastern area of Harris County. Also, a geospatial association between the presence of TRO around the schools and current use of e-cigarettes was observed in the entire Tarrant County and in the northeastern area of Harris County.

**Conclusions:**

There were geospatial associations between the presence of TRO around some schools and cigarette/e-cigarette use among students, but this association was not consistent across all the counties. More research is needed to determine why some areas are at higher risk for this association.

## Introduction

Tobacco use remains an enormous public health burden and the leading cause of preventable death around the globe [1]. Among youth, tobacco use is particularly problematic as this behavior increases the chances of addiction and continued use as adults [2–4]. With increased awareness of the dangers of tobacco use and sustained efforts to curtail smoking through local, state, and national policies, the prevalence of cigarette smoking has reduced over the past 20 years, yet many have started to use other forms of tobacco like e-cigarettes. e-Cigarettes have the potential for nicotine toxicity due to high levels of nicotine in the cartridges [5]. Comprehensive data on the long-term health effects of e-cigarettes use are currently unavailable.

Recently, several studies have examined the prevalence of cigarette and e-cigarette use in the U.S. The Population Assessment of Tobacco and Health (PATH) study in 2013–2014 assessed a representative sample of youth 12–17 years old in the U.S. [6,7]. PATH reported that while 13.4% and 10.7% were ever users of cigarettes and e-cigarettes, respectively, 4.6% and 3.1% of participants were past 30-day users. During the academic year of 2014–2015, the Texas Adolescent Tobacco and Marketing Surveillance System (TATAMS) examined a representative sample of students enrolled in 6^th^, 8^th^ and 10^th^ grades [8]. TATAMS reported that 10.9% and 19.5% were ever users while 3.5% and 7.4% were past 30-day users of cigarettes and e-cigarettes respectively. TATAMS also had a higher prevalence of current use of cigarette (5.3%) and e-cigarette (10.6%) than PATH after the age-standardization to the PATH population.

One of the dominant channels for tobacco advertising in the U.S. is at Tobacco Retail Outlets (TRO) (e.g., ads posted at the retail location). Tobacco advertising and promotional activities at TROs motivate young people to initiate cigarette use as the advertisements create positive impressions and attitudes towards smoking [3,9]. Studies examining TRO activities in US retail outlets have reported higher cigarette marketing in stores that are more frequently visited by adolescents as opposed to those stores less frequently visited [9,10]. In 2003, a longitudinal study of three middle schools in California found that exposure to retail cigarette advertising was a risk factor for initiating cigarette use, susceptibility to cigarette use and smoking status [11]. Recently, recall of e-cigarette advertisements at TRO was significantly associated with adolescent e-cigarette susceptibility and use in a longitudinal study [12]. However, there is currently limited information on the association between the presence of TRO advertisement around schools and current use of e-cigarettes by youths in the U.S. One study looked at the association of TRO and current use of cigarettes but only in 3 middle schools in California [9]. Though certain studies have utilized Geographic Information Systems in describing the density of tobacco retail outlets, they did not examine how it influences tobacco use behavior [13–15]. Few studies have linked the presence and density of the outlets with cigarette use behavior but none has done so with e-cigarettes [16,17].

The aim of the study is to examine the association of the presence of TRO around schools on adolescents’ current use of cigarettes and e-cigarettes among the 2014–2015 TATAMS representative sample of students enrolled in 6^th^, 8^th^ and 10^th^ grades in Texas. We conducted a secondary data analysis by county and our hypothesis was that students attending schools surrounded by TRO selling tobacco would have higher prevalence of current cigarette and e-cigarette use.

## Methods

### Study design, participants and study areas

**Figure 1:**
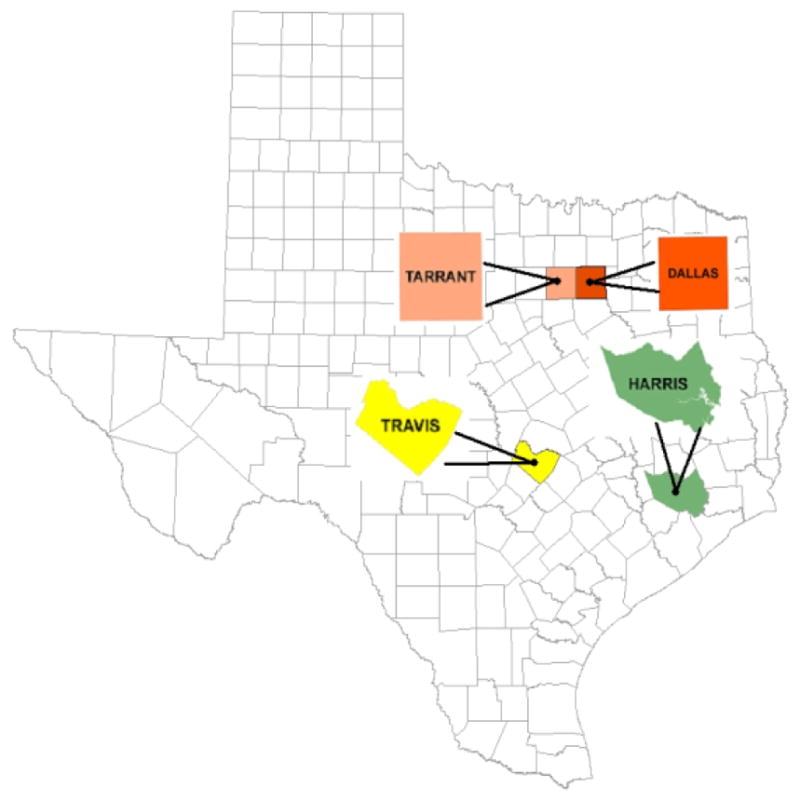
The analyses are for three county areas.

The details of the TATAMS sampling design, sampling frame, and sampling weights were described previously with a summary presented here. TATAMS used a complex random sample of students enrolled in grades 6, 8 and 10th from five counties in Texas (Harris, Dallas/Tarrant, Bexar, and Travis) that surround the four largest metropolitan areas in Texas (Houston, Dallas/Fort Worth, San Antonio, and Austin) [8]. The sample of schools (n=5) were too few to conduct a geospatial analysis in Bexar County. Schools and surrounding TRO in Dallas and Tarrant counties were collapsed for this geospatial analysis given that they are geographically side by side. The analyses are for three county areas (Figure 1). In 2014–2015, the weighted sample was 49% female; 54.8% Hispanic, 21.4% non-Hispanic White, 17.2% non-Hispanic Black; 6.6% Other race/ethnicities; 18.3% with family standard of living as just getting by to poor [8]. The University of Texas Health Science Center at Houston’s Institutional Review board approved this study (#HSC-SPH-13-0377).

### Measures

The list of permitted tobacco retail outlets (TRO), was obtained in November 2014 from the Texas Comptroller of Public Accounts (2014) [18]. The number of TRO within a half-mile radius around each school served to identify two strata: schools without TRO and schools with one or more TRO. Twenty five percent of the participants were located in schools without TRO [8]. Students were identified as current cigarette users if they answered ‘Yes’ to the question ‘Have you ever tried cigarette smoking, even one or two puffs?’ and responded that the number of days were greater than 0 to the question ‘During the past 30 days, on how many days did you smoke cigarettes?’. Students were identified as current e-cigarette users if they answered ‘Yes’ to the question ‘Have you ever tried electronic cigarette, vape pen, or e-hookah, even one or two puffs?’ and reported the number of days as greater than 0 in the question ‘During the past 30 days, on how many days did you smoke electronic cigarette, vape pen, and e-hookah?’. Students were asked “During the past 30 days, how often have you visited the following places near your school?” for stores: “Gas station, convenience/corner stores”, “Drug stores such as Walgreens” and “Grocery stores”. If a student responded “never” to visiting all three stores, then he/she was classified as never visiting any of these stores near his/her school, otherwise he/she was classified as visiting at least one of the stores. If students visited any of these places, then they were asked “When you visited [stores], how often did you see [signs]?” with signs described as (i) marketing cigarettes, marketing electronic cigarettes, vape pens, or e-hookah”, and (iii) “warning about the dangers of smoking (not including warnings on packages)”. Responses were collapsed into two categories “never/not that I remember” versus “recall any signs marketing cigarettes”, “recall any signs marketing e-cigarettes”, and “recall any warning signs”, respectively. Additional control variables included: sex, race/ethnicity, grade, family standard of living, and three school zip-code level characteristics from the 2014 American Community Survey 5 Year Estimates [19], covering 2010 to 2014, including (i) percentage of high school graduate or higher, (ii) the median household income in the past 12 months in 2014 inflation adjusted dollars, and (iii) the percent below poverty level for the population for whom poverty status is determined.

### Statistical analysis

All analyses use sampling weights to account for the complex sampling design. Differences in sociodemographic characteristics for current use of cigarette and e-cigarette users were estimated using chi-square statistics. We applied a Bayesian structured additive regression model to carry out spatial analyses [20]. A spatial function was included by using the Markov random fields, known as the structured additive regression (STAR) model [21] with an intrinsic conditional autoregressive prior. The Markov chain Monte Carlo simulation method was used for estimating the unknown parameters in this model. One spatial model investigated if there was an association between the presence or absence of TRO near the schools and the prevalence of current use of cigarettes and another for e-cigarettes. An interaction of the spatial function with TRO was included after adjusting for sex, race/ethnicity, grade, family standard of living, reported visiting stores near the school during past 30 days, recalling signs marketing cigarettes (for the cigarette model) or recallin signs marketing e-cigarettes (for the e-cigarette model), recalling warning signs, the percentage of high school graduates or higher in the school zip code, the median household income in the school zip code, and the percent below poverty level from the school zip code. Each model was estimated for each study area. This is a total of three geospatial models for cigarette use and another three for e-cigarette use.

The regression model can be represented in the following way. In statistical terms, suppose the response Y_ij_ represents current use of cigarettes. The subscriptions (i, j) indicate the student index i and the school index j. Equation 1 describes the STAR model building a Bayesian geoadditive logistic model framework.

(1) $$\mathrm{Logit}\left(Y_{ij}\right)=\alpha +\beta X_{i}+\gamma Z_{j}+\left({\mathrm{TRO}}_{j}\right)\times f_{\mathrm{spat}}(j)$$

where Logit(.) is a logit function for log[P(Y_ij_=1)/(1−P(Y_ij_=1)], the unknown parameter α indicates a fixed intercept, β identifies a 6×1 vector containing six unknown parameters for individual-level confounders **X_i_** (i.e., sex, race/ethnicity, grade, family standard of living, recalling marketing signs, recalling warning signs), γ identifies a 3×1 vector containing three unknown parameters for socioeconomic status variables **Z_j_** (i.e., the percentage of high school graduate or higher, the median household income, and the percent below poverty level) from zip codes where school j is located. TRO_j_ is a dummy variable of whether there is at least one TRO around school j, and it interacts with a spatial function f_spat_(j), which is the Markov random fields taking spatial autocorrelation into account [22,23]. Further, all estimated coefficients were weighted by the reciprocal of the number of students in each school. Adjusted odds ratios (OR) were calculated for **X_i_** and **Z_j_** from exponential estimates of β and γ. The spatial function generated a spatial estimate in each school. Those spatial estimates are transformed into relative risks (RR) of the presence of the TRO on the current use of cigarettes or e-cigarettes in a school compared to all schools. Then, for estimating whether the other non-selected schools have a potential risk of higher students’ current use of cigarettes or e-cigarettes, we applied a univariate ordinary Kriging method to interpolate values among the rest of the schools in each county, and conducted a kriged map to show hot-spots inside the boundary of each study areas. All kriged values are presented in terms of the color patterns of HSV (hue, saturation and value) from blue color (Smaller RR) to red (Higher RR). Hence a hot-spot can be easily identified from those areas with a kriged RR shown in red color. We also add black dots in the maps representing the locations of participating schools.

TATAMS in 2014–2015 surveyed 3,907 students and 142 were excluded who were located on Bexar County. For the models regarding current use of cigarettes, 24 were excluded because they did not report their family standard of living or current use of cigarettes. For the models regarding current use of e-cigarettes, 21 students were excluded because they did not report their family standard of living or current use of e-cigarettes. Because of the low prevalence of current users of cigarettes in Harris county and visiting stores near schools during past 30 days, this covariate was excluded from this county model with the purpose to obtain convergence. This happened similarly for current use of cigarettes in Travis county and race/ethnicity that were excluded as covariates with the purpose of obtaining convergence in Travis county. Data management and demographic analyses were accomplished by SAS v9.4 (SAS Institute Inc., Cary). Geospatial analyses were implemented by R2BayesX package in R v3.2.4 and Spacestat 4.0. Maps were drawn by ArcGIS (ERSI) and Spacestat 4.0. We used a type I error level of 0.05.

## Results

This study included students from 32, 20 and 22 schools in Dallas/Tarrant, Harris, and Travis counties, respectively. Table 1 provides descriptive statistics for current users and non-current users of cigarettes and e-cigarettes in the study areas. Current users of cigarettes or e-cigarettes were more likely to be 10^th^ graders and have a family standard of living as very well off, respectively. The influence of covariates on current use of cigarettes and e-cigarettes by each study area is shown in Table 2. In Harris and Travis counties, students in 8^th^ and 10^th^ grades had higher odds than 6^th^ grade students of current use of cigarettes. The adjusted odds of current use of cigarettes for students who reported that their families were just getting by or were poor as their standard of living in Dallas/Tarrant county were higher as compared to those living comfortably. In Dallas/Tarrant and Travis counties, students in 8^th^ and 10^th^ grades had higher odds than 6^th^ grade students of current use of e-cigarettes. In Harris and Travis counties, the odds of current use of e-cigarettes were higher among those who recalled sings marketing signs e-cigarette in stores around their school in comparison to those students who do not recall signs marketing signs of e-cigarettes in stores around their school, after adjusting for covariates.

**Figure 2:**
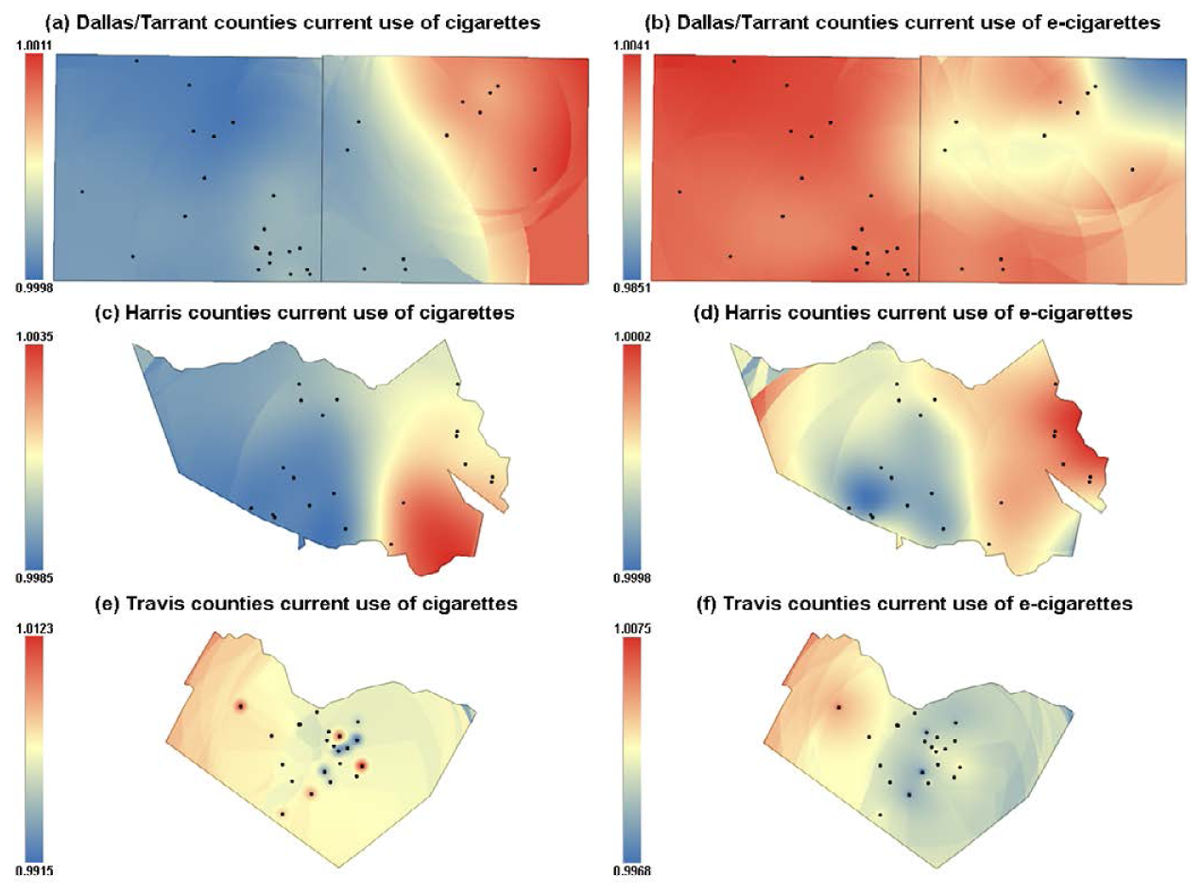
The association of the geospatial presence of the TRO on current use of cigarettes (panel a) and e-cigarettes (panel b) by each study area, Dallas/Tarrant, Harris and Travis counties.

Figure 2 shows the association of the geospatial presence of the TRO on current use of cigarettes (panel a) and e-cigarettes (panel b) by each study area, Dallas/Tarrant, Harris and Travis counties, respectively. The blue areas on the left of panel a indicate that there is not an association between the geospatial presence of the TRO and current use of cigarettes in Tarrant county. On the contrary, the red areas on the left of panel a indicate that there is an association in the geospatial presence of the TRO on current use of cigarette in the eastern area of Dallas county where there are five schools in that hot-spot. We observed an association in the geospatial presence of the TRO on current use of e-cigarettes in the entire Tarrant county, particularly in the north and northwestern areas (panel b). Two participating schools were located nearby the hot-spot of the geospatial presence of the TRO and current use of cigarettes in the southeastern area of Harris county (panel c). Panel d also shows that in eastern Harris county there is an association in the geospatial presence of the TRO and current use of e-cigarettes. Six study schools were surrounded by TRO in the hot-spot of current use of e-cigarette in the northeastern area of Harris county (panel d). Panels e and f do not present a clear pattern of associations of the presence of TRO with current use of cigarettes or e-cigarettes in Travis county.

## Discussion

To our knowledge this is the first study to report the geospatial association of the presence of tobacco retail outlets within a half-mile radius around schools with current use of e-cigarettes. Other studies have examined this relationship with cigarette smoking [9,17]. By design, the majority of the schools in this study (75%) had at least one to TRO within a half-mile radius which represented the proportion of TRO in their sampling frame for each county [8]. Students in schools in hot-spots of Dallas/Tarrant and Harris counties, who had TRO around their schools, had higher RR of current use of cigarettes or e-cigarettes after adjusting for multiple covariates. This finding is intuitive and supports the hypothesis that tobacco use behavior is influenced by the density of tobacco retail outlets around where they live or study [16,17]. The easy access to the tobacco products, less retrieval cost and exposure to more brand promotions and/or tobacco advertising may be encouraging use [24] in these hot-spots identified. Policies and interventions that lead to the reduction in the number of TRO around the schools in the hot-spots may help in reducing the use of tobacco products by adolescents [25].

Exploring the potential for limiting the density and types of TRO, as well as increasing the distance from the TRO to the schools may be a plausible strategy to reduce the increasing prevalence of e-cigarette use [16]. The potential of such policies will need to be explored and evaluated. There are several policy research challenges that will need to be addressed in future studies [15]. First, is there an appropriate number of cigarette and e-cigarette advertisements per TRO that should be permitted around the schools?. Past studies have shown that there are more exterior cigarette ads near schools and more ads where kids shop [14,26], but to our knowledge we do not know of any study on e-cigarette ads. Second, the U.S. Food and Drug Administration (FDA) has been granted specific regulatory authority to restrict the manufacturing, distribution, and marketing of tobacco products [27]. Enforcing any FDA advertising regulations will have a significant number of challengers not only to understand how the states or counties implement such policies, but the crucial component of providing adequate and reliable scientific research that links TRO advertisements with tobacco use behaviors by adolescents. Third, the impact of these policies need to be evaluated. In California, for example, 31% of the TRO at the start of the intervention stopped marketing tobacco post-intervention [13], demonstrating that it is feasible to evaluate interventions at TRO. However, evaluating such interventions will need to be, like the current study, linked with tobacco use behaviors across time.

The strength of this study is the use of geospatial modeling to consider the geographic association of current use of cigarette and e-cigarette and presence of TRO near schools. The results of interpolation can provide further information to schools that were not selected at random, especially in Harris and Dallas/Tarrant counties. Some limitations existed in this study. First, schools were selected based on the sampling design of TATAMS and not by the geographic distribution of schools across the county, and as such, are not uniform. Second, some counties had some areas without enough data to support the findings, even after using kriging, such as the southeastern Dallas, western Harris, and eastern of Travis counties. Third, the number of TRO may vary over time, while our data cannot reflect the variation, we considered whether schools have at least one TRO reducing potential bias from varied TRO. The sample size for current use of cigarettes was not enough in Harris and Travis, causing insufficient data to estimate associations in youth in those populations.

## Conclusions

The identification of hot-spots in the Texas counties, where the presence of TRO is associated with cigarette or e-cigarette use is important, as these findings support the potential need for regulation of TRO around the identified geospatial areas. More research on what constitutes a hot-spot is warranted.

## Figures and Tables

**Table 1:** Summary statistics of current use of cigarettes and e-cigarettes in TATAMS in 2014–2015 overall counties.

Variable	Cigarette				E-Cigarette			
	Current user		Non-current user		Current user		Non-current user	
	n=80 (N=14249)		n=3661 (N=418014)		n=245 (N=31126)		n=3499 (N=401293)	
	n (N)	%	n (N)	%	n (N)	%	n (N)	%
Sex								
Boys	41 (7159)	50.2	1609 (216363)	51.8	134 (17018)	54.7	1516 (206610)	51.5
Girls	39 (7092)	49.8	2052 (201651)	48.2	111 (14108)	45.3	1983 (194683)	48.5
Race/Ethnicity								
Black	11 (2384)	16.7	604 (76273)	18.2	46 (4855)	15.6	567 (73366)	18.3
Hispanic	43 (9179)	64.4	1319 (216850)	51.9	113 (18983)	61.0	1256 (207737)	51.8
White/Other	26 (2685)	18.9	1738 (124891)	29.9	86 (7288)	23.4	1676 (120190)	29.9
Grade	**				***			
6th	6 (1208)	*8.5*	1099 (139692)	*33.4*	*15 (2938)*	*9.4*	*1093 (138061)*	*34.4*
8th	17 (3932)	*27.6*	1217 (140863)	*33.7*	*54 (8612)*	*27.7*	*1180 (136348)*	*33.9*
10th	57 (9109)	*63.9*	1345 (137458)	*32.9*	*176 (19575)*	*62.9*	*1226 (126883)*	*31.7*
Tobacco Retail Outlets**								
+1	57 (12772)	*89.6*	1831 (105860)	*74.7*	148 (25023)	80.4	1740 (299922)	74.7
0	23 (1477)	*10.4*	1830 (312154)	*25.3*	97 (6103)	19.6	1759 (101370)	25.3
Family’s standard of living***					**			
Living comfortably	*8 (805)*	*5.7*	*849 (86801)*	*20.8*	*45 (5228)*	*16.8*	*813 (82732)*	*20.6*
Very well off	*44 (7865)*	*55.2*	*2280 (260783)*	*62.4*	*140 (17391)*	*55.9*	*2182 (250778)*	*62.5*
Just getting by-poor^a^	*28 (5579)*	*39.1*	*532 (70431)*	*16.8*	*60 (8506)*	*27.3*	*504 (67783)*	*16.9*
During past 30 days visited stores near school								
Yes	76 (14006)	98.3	3600 (412445)	98.7	238 (30498)	98.0	3442 (396147)	98.7
No	4 (243)	1.7	61 (5569)	1.3	7 (628)	2.0	57 (5146)	1.3
Recall any warning signs about the dangers of smoking								
Yes	37 (6449)	45.3	2093 (237640)	56.8	136 (16056)	51.6	2001 (228706)	57.0
No	43 (7800)	54.7	1568 (180373)	43.2	109 (15069)	48.4	1498 (172587)	43.0
Recall any signs marketing cigarettes					Recall any signs marketing e-cigarettes**			
Yes	65 (11962)	83.9	3146 (345128)	82.6	*156 (20878)*	*67.1*	*1742 (200482)*	*50.0*
No	15 (2287)	16.1	515 (72886)	17.4	*89 (10248)*	*32.9*	*1757 (200811)*	*50.0*

n=sample size, N=weighted sample size;

a Family’s standard of living as “just getting by”, “nearly poor,” or “poor.”

**Note:** Italicized values indicates Statistical significance (*p<0.05, **p<0.01, ***p<0.0001).

**Table 2:** Adjusted odds ratio of linear predictors for current use of cigarettes and e-cigarettes by county.

	Dallas/Tarrant		Harris		Travis	
Variable	AOR (95%CI)	Sig	AOR (95%CI)	Sig	AOR (95%CI)	Sig
Current Use of Cigarettes						
Sex						
Boys	1.15 (0.56–2.37)		1.45 (0.28–7.42)		0.75 (0.29–1.97)	
Girls	1					
Ethnicity						
Black	1		1		NA	
Hispanic	2.04 (0.66–6.36)		1.59 (0.40–6.32)			
White/Other	2.44 (0.77–7.66)		1.79 (0.58–5.47)			
Grade						
6^th^	1					
8^th^	0.42 (0.06–3.17)		*3.45 (1.51–7.89)*	**	*5.47 (1.01–29.48)*	**
10^th^	3.18 (0.91–11.06)		*3.13 (2.58–3.81)*	**	*12.85 (2.029–81.36)*	**
Family’s standard of living						
Living comfortably	1		1		1	
Very well off	2.22 (0.48–10.21)		1.73 (0.7–4.50)		1.81 (0.38–8.73)	
Just getting by-poor^a^	*8.26 (1.77–38.60)*	**	1.97 (0.32–12.08)		3.59 (0.70–18.34)	
Recall any signs marketing cigarettes						
No	1		1			
Yes	0.86 (0.30–2.46)		1.56 (0.29–8.48)		1.59 (0.33–7.60)	
Recall any warning signs about the dangers of smoking						
No	1		1		1	
Yes	0.65 (0.31–1.37)		1.45 (1.00–2.09)		1.38 (0.51–3.78)	
Current use of e-cigarettes						
Sex						
Boys	1.24 (0.84–1.84)		0.69 (0.45–1.01)		1.64 (0.92–2.95)	
Girls	1					
Ethnicity						
Black	1		1		1	
Hispanic	1.13 (0.67–1.90)		0.89 (0.52–1.50)		2.45 (0.32–18.71)	
White/Other	1.58 (0.93–2.66)		0.62 (0.32–1.22)		4.95 (0.62–39.43)	
Grade						
6^th^	1					
8^th^	*4.62 (1.57–13.57)*	**	1.22 (0.36–4.13)		*4.95 (1.56–15.76)*	**
10^th^	*10.81 (4.41–26.45)*	**	2.83 (0.95–8.40)		*22.62 (7.17–71.43)*	**
Family’s standard of living						
Living comfortably	1		1		1	
Very well off	0.70 (0.42–1.17)		1.12 (0.62–2.03)		1.03 (0.49–2.18)	
Just getting by-poor^a^	1.32 (0.75–2.33)		1.13 (0.54–2.34)		0.99 (0.41–2.39)	
Recall any signs marketing e-cigarettes						
No	1		1			
Yes	1.31 (0.88–1.96)		*1.72 (1.11–2.68)*	**	*2.35 (1.22–4.55)*	**
Recall any warning signs about the dangers of smoking						
No	1		1		1	
Yes	0.97 (0.66–1.42)		1.08 (0.70–1.65)		1.42 (0.78–2.58)	

**Note:** Italicized values indicates statistical significance (*p<0.05, **p<0.01, p<0.0001). Adjusted for during past 30 days visited stores near school, the percentage of high school graduate or higher in the school zip code, the median household income in the school zip code, and the percent below poverty level from the school zip code.

a Family’s standard of living as “just getting by”, “nearly poor,” or “poor.”

AOR (95%CI), Adjusted odds ratios (OR) with 95% credible interval (CI).
